# In Vitro SARS-CoV-2 Infection of Microvascular Endothelial Cells: Effect on Pro-Inflammatory Cytokine and Chemokine Release

**DOI:** 10.3390/ijms23074063

**Published:** 2022-04-06

**Authors:** Maria Dolci, Lucia Signorini, Sarah D’Alessandro, Federica Perego, Silvia Parapini, Michele Sommariva, Donatella Taramelli, Pasquale Ferrante, Nicoletta Basilico, Serena Delbue

**Affiliations:** 1 Department of Biomedical, Surgical and Dental Sciences, University of Milan, Via Pascal 36, 20133 Milan, Italy; maria.dolci@unimi.it (M.D.); lucia.signorini@unimi.it (L.S.); federica.perego@unimi.it (F.P.); pasquale.ferrante@unimi.it (P.F.); serena.delbue@unimi.it (S.D.); 2 Department of Pharmacological and Biomolecular Sciences, University of Milan, Via Pascal 36, 20133 Milan, Italy; sarah.dalessandro@unimi.it (S.D.); donatella.taramelli@unimi.it (D.T.); 3 Department of Biomedical Sciences for Health, University of Milan, Via Pascal 36, 20133 Milan, Italy; silvia.parapini@unimi.it; 4 Department of Biomedical Sciences for Health, University of Milan, Milan, Mangiagalli 31, 20133 Milan, Italy; michele.sommariva@unimi.it

**Keywords:** Human Microvascular Endothelial Cells (HMEC-1), SARS-CoV-2, inflammatory mediators

## Abstract

In the novel pandemic of Coronavirus Disease 2019, high levels of pro-inflammatory cytokines lead to endothelial activation and dysfunction, promoting a pro-coagulative state, thrombotic events, and microvasculature injuries. The aim of the present work was to investigate the effect of SARS-CoV-2 on pro-inflammatory cytokines, tissue factor, and chemokine release, with Human Microvascular Endothelial Cells (HMEC-1). ACE2 receptor expression was evaluated by western blot analysis. SARS-CoV-2 infection was assessed by one-step RT-PCR until 7 days post-infection (p.i.), and by Transmission Electron Microscopy (TEM). IL-6, TNF-α, IL-8, IFN-α, and hTF mRNA expression levels were detected by RT-PCR, while cytokine release was evaluated by ELISA. HMEC-1 expressed ACE2 receptor and SARS-CoV-2 infection showed a constant viral load. TEM analysis showed virions localized in the cytoplasm. Expression of IL-6 at 24 h and IFN-α mRNA at 24 h and 48 h p.i. was higher in infected than uninfected HMEC-1 (*p* < 0.05). IL-6 levels were significantly higher in supernatants from infected HMEC-1 (*p* < 0.001) at 24 h, 48 h, and 72 h p.i., while IL-8 levels were significantly lower at 24 h p.i. (*p* < 0.001). These data indicate that in vitro microvascular endothelial cells are susceptible to SARS-CoV-2 infection but slightly contribute to viral amplification. However, SARS-CoV-2 infection might trigger the increase of pro-inflammatory mediators.

## 1. Introduction

Coronavirus disease 2019 (COVID-19) is a rapidly escalating world pandemic, caused by Severe Acute Respiratory Syndrome CoronaVirus-2 (SARS-CoV-2) infection. SARS-CoV-2 accesses host cells via the protein angiotensin-converting enzyme 2 (ACE2) receptors, which is abundantly expressed in the lungs and in other human tissues, such as the endothelium [1,2,3]. Endothelial cells (EC) play a crucial role in several physiological processes, such as the regulation of vascular tone, blood fluidity, and the production of inflammatory and immunological mediators [4]. However, it remains unclear whether SARS-CoV-2 can directly infect the endothelium [5,6]. The major clinical events usually observed in COVID-19 patients, such thrombosis and cerebrovascular disorders, indicate that the virus is targeting the endothelium [7]. The pathological steps from SARS-CoV-2 infection and the COVID-19 disease can be divided into three phases: (i) an asymptomatic phase; (ii) a non-severe symptomatic phase with upper airway involvement; and (iii) a severe disease with hypoxia, cellular infiltrates in the lung, and progression to acute respiratory distress syndrome (ARDS). In the latter phase, the viral replication in the lung parenchyma causes severe interstitial inflammation and triggers an abundant production of pro-inflammatory cytokines and chemokines called “cytokine storm” [8,9]. In addition, cardiovascular complications are rapidly emerging as a key threat in COVID-19 pathogenesis [5]. Increased level of pro-inflammatory cytokines and a decrease of anti-inflammatory cytokines was detected in the peripheral blood of COVID-19 patients [10]. Furthermore, the clinical demonstration of coagulation anomalies, interferon release, virus-induced vascular damage, and secondary ischemia clearly supports the hypothesis that widespread endothelial infection could have a key pathogenetic role in the severe forms of COVID-19 [11,12]. Finally, histological analysis of biopsies of infected patients demonstrated vascular damage and endothelium apoptosis, supporting a causal relation with SARS-CoV-2 infection [5,11]. The aim of the present study was to verify whether SARS-CoV-2 infection in Human Microvascular Endothelial Cells (HMEC-1) might be involved in the host pro-inflammatory response to the virus.

## 2. Results

### 2.1. HMEC-1 Cells Express Low Levels of ACE2 

**Figure 1:**
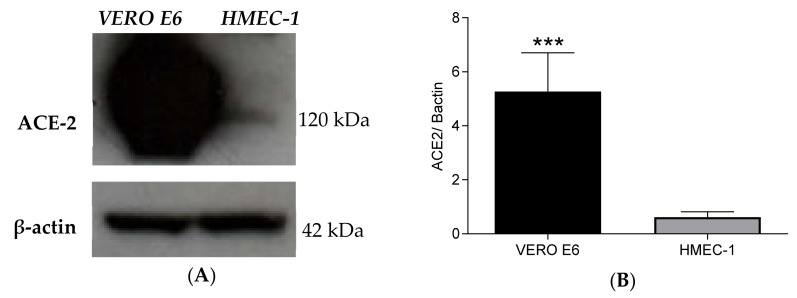
Detection of ACE2 protein in VERO E6 and HMEC-1 cells. (**A**) Western blot analysis showing ACE2 (about 120 kDalton) in VERO E6 and HMEC-1 cell lines. (**B**) Quantification of ACE2 intensity. ACE2 expression was normalized versus β-actin expression. Statistical significance was determined using *t*-test, between HMEC-1 and VERO E6 (*** *p* value < 0.0001).

Since SARS-CoV-2 infects the host cells using ACE2 receptors, the expression of ACE2 was determined in HMEC-1. VERO E6 cells, notably susceptible to SARS-CoV-2 infection, were used as control. Western blot analysis confirmed the ACE2 protein expression in both VERO E6 and in HMEC-1 (Figure 1); however, the expression of ACE2 protein was drastically lower in HMEC-1 compared to VERO E6 cells (*** *p* < 0.0001).

### 2.2. HMEC-1 and VERO E6 SARS-CoV-2 Infection

**Figure 2:**
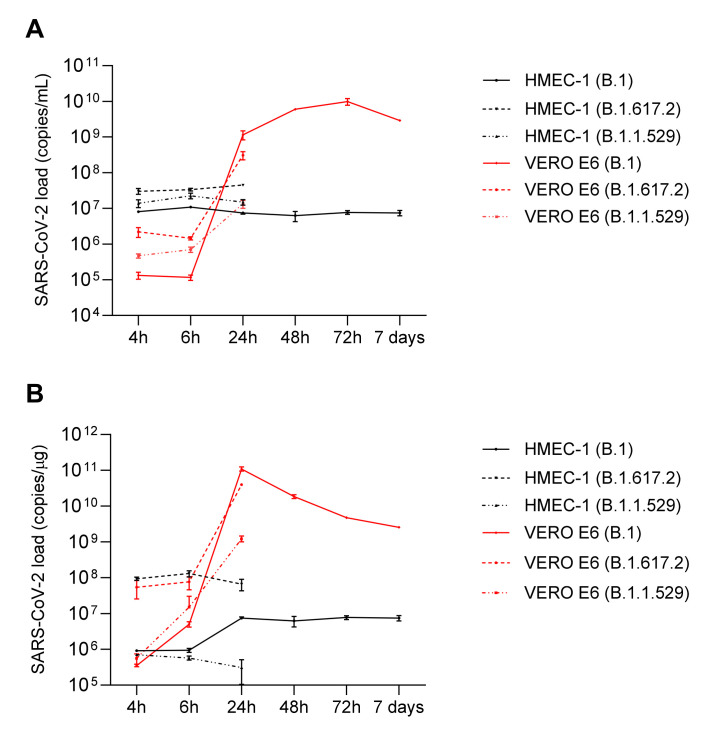
SARS-CoV-2 variants growth curves in HMEC-1 and VERO E6 cell lines. At 4, 6, 24, 48, 72 h and 7 days p.i., SARS-CoV-2 N1 gene expression in cell supernatants (panel (**A**): viral load expressed as copies/mL) and cellular RNA (panel (**B**): viral load expressed as copies/µg) of infected HMEC-1 and VERO E6 cell lines was evaluated by means of qRT-PCR.

**Figure 3:**
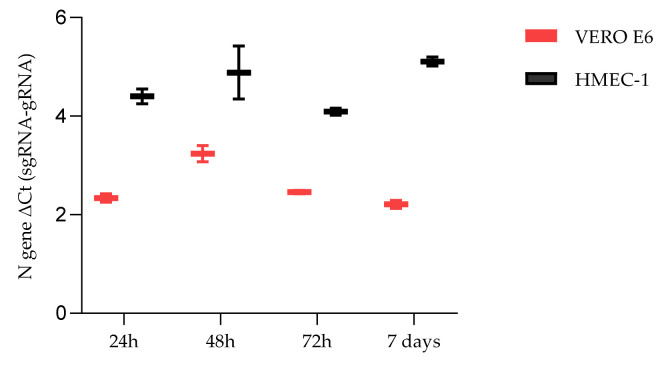
SARS-CoV-2 variant B.1 N1 gene ΔCt (sgRNA-gRNA) values. The sgRNA and gRNA cycle threshold values at 24, 48, 72 h and 7 days p.i. were obtained by means of two qRT-PCRs targeting the N1 gene. Plots whiskers indicate down to the minimum and up to the maximum value for each time point.

Growth curves of the SARS-CoV-2 viral strains B.1 (SARS-CoV-2/human/ITA/Milan-UNIMI-1/2020, GenBank MT748758.1), B.1.617.2 (GISAID EPI_ISL_6602889), B.1.1.529, and BA.1 (EPI_ISL_10898045) were defined through infecting HMEC-1 cells and quantifying the viral load by RT-qPCR in the cell supernatants and cellular RNA extracted from infected cells at 4, 6, 24, 48, 72 h and 7 days p.i. for the B.1 strain and 4, 6, and 24 h for the B.1.617.2 and B.1.1.529 strains (Figure 2). Growth curves were compared with those obtained infecting VERO E6 cells. As for HMEC-1, the viral load was constant at each time point p.i. both in the supernatants and in the cells. In contrast, in the VERO E6 cells, the production of SARS-CoV-2 virions increased from 4 h to 72 h p.i., while a decreasing trend was observed in cellular load from 24 h to 7 days p.i. The viral load in supernatants and cellular RNA of HMEC-1 and VERO E6 cells, at each time point, is reported in the Table S1 (Supplementary). These data suggest that SARS-CoV-2 enters HMEC-1, but its replication is not robustly supported. Furthermore, detection of SARS-CoV-2 sgRNA of the N gene was performed in both cell lines and for each viral strain. Representative results obtained for the B.1 strain are shown in Figure 3. Calculation of ΔCt between gRNA Ct and the corresponding sgRNA Ct was performed at each time point (Table S2). Although the gRNA levels were higher compared to sgRNA at each time point p.i., the presence of sgRNA demonstrated that SARS-CoV-2 can also minimally replicate in HMEC-1. 

### 2.3. SARS-CoV-2 Localization in HMEC-1

**Figure 4:**
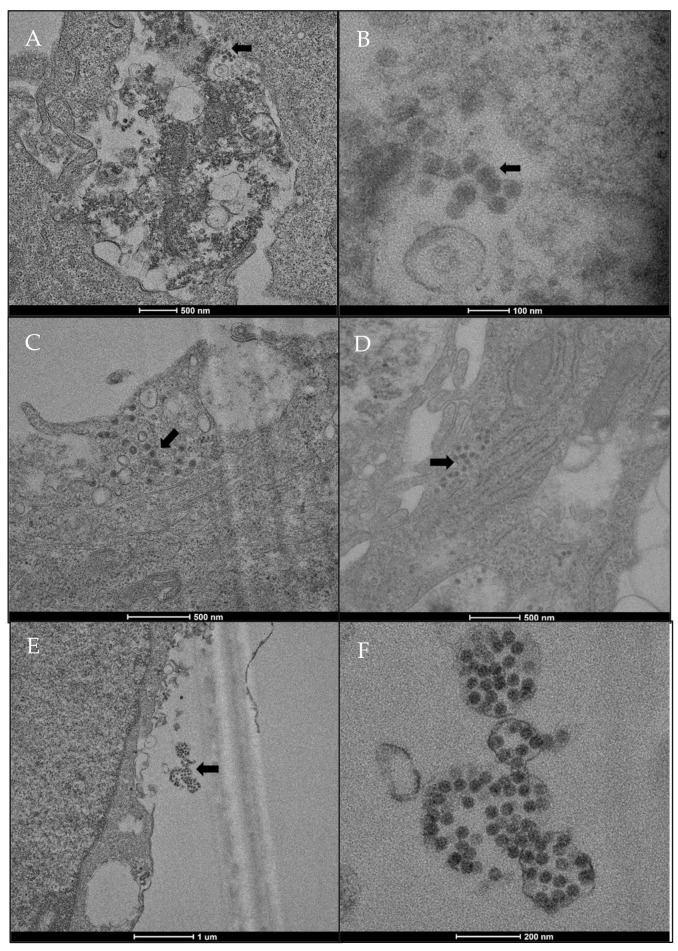
Localization of SARS-CoV-2 in HMEC-1 cell line, analyzed by TEM. (**A**) HMEC-1 presenting SARS-CoV-2 infection (arrow) and a severe degeneration (scale bar: 500 nm). (**B**) Enlargement of previous figure, showing spherical viral electron-dense particles within vacuoles (scale bar: 100 nm). (**C**) Spherical viral particles located in the cytosol (scale bar: 500 nm). (**D**) Spherical viral particles located in the cytosol, near the RER. (**E**) Aggregate of spherical viral particles outside the cells (scale bar: 1 µm). (**F**) Enlargement of panel (**E**), showing spherical viral particles, containing black dots on the inside, within structures with smooth membrane (scale bar: 200 nm).

Figure 4 shows the viral particles of SARS-CoV-2—as electron-dense corpuscles, localized in different compartments of the HMEC-1 cells—appearing severely degenerated due to the infection (panels A and B). SARS-CoV-2 virions were detected in the cytosol (panel C) and near the Rough Endoplasmic Reticulum (RER) (panel D), and outside of cells, appearing as dense corpuscles inside membranous structures (panels E and F).

### 2.4. Cytokines and Tissue Factor Transcription upon SARS-CoV-2 HMEC-1 Infection

**Figure 5:**
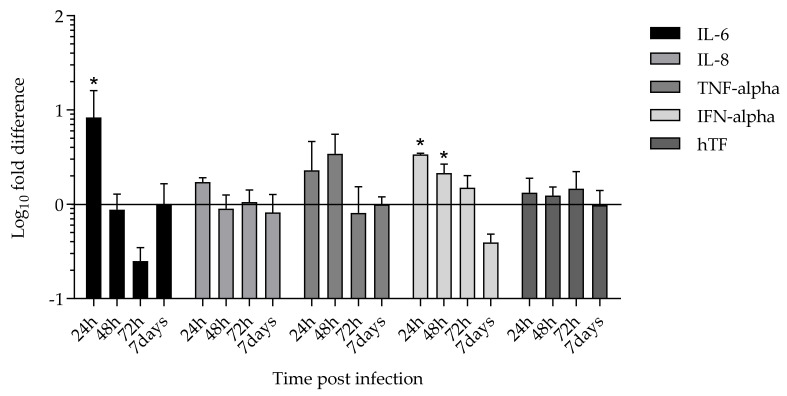
IL-6, IL-8, TNF-α, IFN-α, and hTF transcription in SARS-CoV-2 B.1 infected HMEC-1. log_10_ fold-change values above 0 express upregulation of target gene. Cytokines and hTF mRNA levels were assessed by reverse transcription, SYBR Green Real Time PCR, and Fold Difference analysis between uninfected and infected cells at 24, 48, 72 h and 7 days p.i. The cytokines and hTF mRNA expression levels were normalized with GAPDH mRNA level and reported in the figure as log_10_ fold difference. Significant difference of IL-6 expression at 24 h and IFN-α at 24 h and 48 h in infected cells compared to uninfected cells was observed; * *p* < 0.05.

The levels of IL-6, IL-8, TNF-α, IFN-α, and hTF transcripts were assessed in infected and uninfected HMEC-1 at 24 h, 48 h, 72 h, and 7 days p.i. (B.1 strain). An increase in IL-6, IL-8, and TNF-α transcripts was detected in the infected compared to non-infected cells at 24 h p.i. (6.3-fold (*p* < 0.05), 1.72-fold, and 2.3-fold, respectively). The level of TNF-α transcript remained higher also after 48 h p.i. (3.10-fold) in SARS-CoV-2 infected cells compared to uninfected HMEC-1. A decreasing trend over time was observed for IFN-α mRNA expression level, which was 3.37-fold higher at 24 h (*p* < 0.05), 2.11-fold higher at 48 h (*p* < 0.05), and 1.47-fold higher at 72 h compared to uninfected HMEC-1. The level of hTF transcript remained constant at all time points (Figure 5). Regarding the B.1.617.2 and B.1.1.529 strain, evaluation of IL-6, IL-8, TNF-α, and IFN-α transcripts at 4 h, 6 h, and 24 h p.i. showed a statistically significant increase at 4 h p.i. for IL-6 for the B.1 and B.1.617.2 strains compared to the non-infected cells.

### 2.5. IL-6, IL-8, TNF-α, and IFN-α Protein Expression 

**Figure 6:**
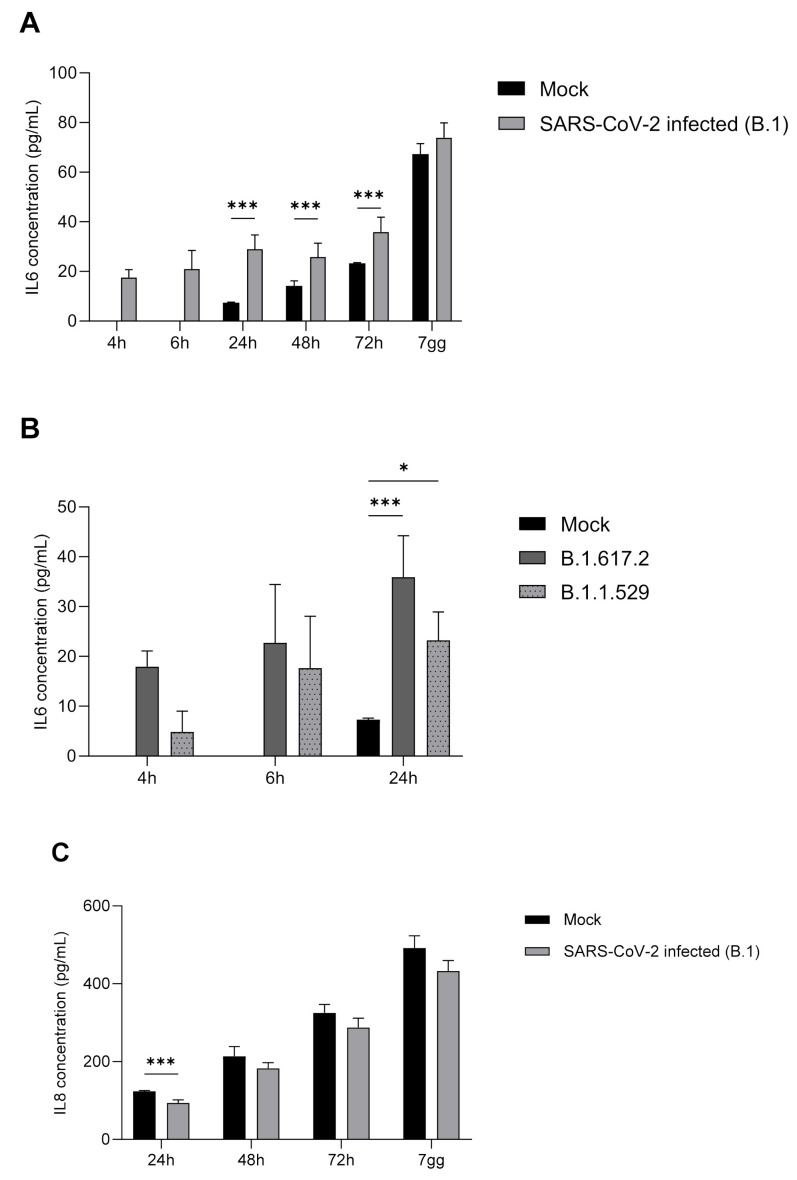
IL-6 and IL-8 evaluation in supernatant of SARS-CoV-2 infected HMEC-1 by ELISA test. IL-6 concentration (pg/mL) in SARS-CoV-2 infected supernatants at 4 h, 6 h, 24 h, 48 h, 72 h, and 7 days p.i. for the B.1 strain (panel (**A**)). Significant IL-6 higher expression was observed in infected cells at 24 h, 48 h, and 72 h p.i. (*** *p* < 0.001) compared to non-infected cells. IL-6 expression at 4 h, 6 h, and 24 h p.i. for the B.1.617.2 and B.1.1.529 strains. Significant IL-6 higher expression was observed at 24 h for both strains (*** *p* < 0.001 and * *p* < 0.05 for B.1.617.2 and B.1.1.529, respectively) (panel (**B**)). IL-8 concentration (pg/mL) in uninfected and SARS-CoV-2 infected supernatants. Significant IL-8 lower expression was observed in infected cells at 24 h p.i.; *** *p* < 0.001 (panel (**C**)).

Spontaneous and time-dependent production of both IL-6 and IL-8 was observed in the supernatants of infected HMEC-1 cells (Figure 6). SARS-CoV-2 infection induced an increase in the extracellular levels of IL-6 compared to uninfected cells, which was statically significant at 24 h, 48 h, and 72 h (Figure 6A), but no longer after 7 days p.i. Similarly, the concentration of IL-8 increased over time in both infected and uninfected cell supernatants (Figure 6C). Surprisingly, the infection with SARS-CoV-2 did not determine a further increase of IL-8 above spontaneous production. Indeed, the levels of IL-8 were lower in culture supernatants of infected cells compared to uninfected control cells at all the time points tested, with a statically significant difference at 24 h (Figure 6C). TNF-α and IFN-α were not detectable in either uninfected or SARS-CoV-2 infected cell supernatants. Spontaneous and time-dependent production of both IL-6 and IL-8 in the supernatants of infected HMEC-1 cells with SARS-CoV-2 B.1, B.1.617.2 and B.1.1.529 strains revealed IL-6 production at 4 h and 6 h p.i. (Figure 6B), but undetectable IL-8 at the same time points. IL-8 was detectable at 24 h p.i. (274.75 pg/mL and 182.02 pg/mL after SARS-CoV-2 B.1.617.2 and B.1.1.529 infections, respectively).

## 3. Discussion

SARS-CoV-2 infection of endothelial cells has been observed both in vivo and in vitro. Infection and replication of SARS-CoV-2 has been observed in human blood capillary organoids [13] and in post-mortem samples of COVID-19 patients [5]; finally, in vitro studies showed non-productive infections of human umbilical vein endothelial cells (HUVECs) and Human Microvascular Endothelial Cells from the lungs (HMVEC-L) [14]. The present study provides further evidence that HMEC-1, a dermal microvascular endothelial cell line, is permissive to SARS-CoV-2 infection, but does not support robust viral replication. However, SARS-CoV-2 infection in HMEC-1 induces the production of pro-inflammatory cytokines. 

Isolation and propagation of SARS-CoV-2 in different cell culture models is widely used in vitro [15]. Specifically, VERO E6 are the common cell type used to support SARS-CoV-2 infection and replication, while no information is available about susceptibility and permissibility of the HMEC-1 cell line. The ACE2 receptor, used by SARS-CoV-2 to infect human cells, is highly expressed in Vero E6 cells and in HMEC-1 cells, as shown here by the means of western blot analysis. These data extend previous observations on endothelial cells of different origin [16,17]. The successful infection of HMEC-1 was confirmed by electron microscopy. Interestingly, SARS-CoV-2 specific RT- qPCR demonstrated the viral entry and persistence in the cells up to 7 days, but a weak production of new viral particles, in line with that shown by Schimmel and colleagues in endothelial cells from lungs and from umbilical vein [14]. It has been shown that in addition to ACE-2, SARS-CoV-2 binds other host cell receptors such as Neuropilin-1 (NRP1) [18,19], a transmembrane protein expressed by many cell types including endothelial cells [20,21,22]. Moreover, TMPRSS2, a protease activator for SARS-CoV-2 entry [23,24], is also expressed on endothelial cells [25]. Binding and interaction of SARS-CoV-2 to these receptors could induce endothelial cell activation and cytokine production. It has indeed been reported that SARS-CoV-2/ACE2 interaction induces cytokine production by macrophages, PBMCs, and epithelial cells [26,27]. While HMEC-1 cells did not robustly replicate SARS-CoV-2, they produced pro-inflammatory cytokines, particularly IL-6. 

Severe infections with SARS-CoV-2 are strongly associated with a cytokine storm characterized by high levels of inflammatory cytokines, including IL-6 and TNF-α, in the plasma of patients [28,29]. The source of this massive production of cytokines has not yet been clarified. IL-6, released during infections or tissue injuries, is one of the main pro-inflammatory cytokines involved in the initiation and amplification of a cytokine storm. High levels of IL-6 are linked to disease severity and poor prognosis in critically ill COVID-19 patients [30]. Here, we have shown that SARS-CoV-2 infection induces the expression of IL-6 by HMEC-1 cells after 24 h from the infection. The amounts of IL-6 released in the cell supernatants was significantly higher in infected cells than in control cells at 24, 48, and 72 h. The increase in IL-6 levels both in controls and infected cells over time reflects the accumulation of cytokines in the cell supernatants. The difference in IL-6 levels in control and in the infected cells observed at 48 and 72 h are probably due to the initial stimulation of the cells by the virus and the induction of the cytokine expression. Furthermore, to consolidate these findings, evaluation of IL-6 levels in cell supernatants infected with SARS-CoV-2 B.1.617.2 and B.1.1.529 strains was performed. A significant production of IL-6 at 24 h p.i. was observed upon infection with both viral strains, compared to non-infected cells. These results were similar to those obtained with the SARS-CoV-2 B.1 strain. It has been shown that SARS-CoV-2 proteins induce the expression and secretion of IL-6 preferentially by monocytes and macrophages, which are indeed the main cells involved in the production of cytokines [31,32]. Our data suggest that endothelial cells may contribute to the high levels of IL-6 observed in the plasma of patients. IL-6 can also act in an autocrine manner on the endothelium, amplifying the production of inflammatory mediators and activating the coagulation cascade [33]. 

TNF-α gene expression was higher in infected cells than in control cells; however, TNF-α protein was not detected in the supernatants. This is not surprising, since there are reports that even LPS can induce the expression of TNF-α mRNA, but not consistent secretion of the protein due to a deficit of TACE, the TNF-α-converting enzyme [34]. Alternatively, HMEC-1 cells may not be sufficiently stimulated by SARS-CoV-2 alone to produce. There are data indicating that TNFα is produced only when more than one stimulus (cytokines or LPS) is added simultaneously to the cells [35]. It cannot be excluded that together with the infection with SARS-CoV-2, additional stimuli—yet to be identified—are needed to induce the secretion of TNF-α from endothelial cells. 

IL-8, a chemokine produced during viral infection, is involved in the recruitment of pro-inflammatory neutrophils, which can contribute to the cytokine storm and inflammation, with consequent tissue injury. Surprisingly, infected cells did not induce the production IL-8 above spontaneous levels, suggesting that microvascular endothelial cells are unlikely to be the source of these cytokines upon infection with SARS-CoV-2.

Similarly to other virus-infected cells, endothelial cells produce type I IFN upon virus infection [36]. In this study, higher expression of INF-α was observed upon viral infection at 24 and 48 h p.i.; however, the protein was not detected in cell supernatants. 

The vascular endothelium is essential for the maintenance of vascular homoeostasis and the control of vascular tone [37]. Tissue factor is a transmembrane protein that binds factor VII, activating the coagulation protease cascade. It is induced on the surface of endothelial cells by different stimuli, including inflammatory cytokines and viruses [38], and thus plays an important role in initiating thrombosis associated with inflammation and infections. A possible role for TF in the thrombosis associated with severe COVID-19 has been proposed [39]. Here, we have shown that the infection alone does not induce the expression of tissue factor, indicating that other mediators are involved in its production by endothelial cells. The present study has established and characterized for the first time an in vitro model of SARS-CoV-2 infection of Human Microvascular Endothelial Cells of dermal origin. This research lays the foundation for the identification of the molecular basis of the inflammatory and coagulant impairment states related to endothelium infection, strengthening the hypothesis that endothelial activation and dysfunctions due to SARS-CoV-2 infection play a key role in severe COVID-19 pathogenesis.

## 4. Materials and Methods

### 4.1. Cell Culture

Human dermal microvascular endothelial cells (HMEC-1) were kindly provided by the Centers for Disease Control and Prevention, Atlanta, GA, USA [40]. HMEC-1 cells were maintained in culture with complete medium composed of MCDB 131 medium (GIBCO-BRL, Scotland) supplemented with 10% fetal calf serum (HyClone, Logan, UT, USA), 10 ng/mL epidermal growth factor (PeproTech, Rocky Hill, NJ, USA), 1 mg/mL hydrocortisone (Sigma Italia, Milan, Italy), 2 mM glutamine (EuroClone, Pero, Italy), 100 U/mL penicillin, 100 mg/mL streptomycin (EuroClone, Pero, Italy), and 20 mM Hepes buffer, pH 7.3 (EuroClone, Pero, Italy).

VERO clone E6 (ATCC CRL-1586™) cells were maintained in culture with Dulbecco’s Modified Eagle Medium (DMEM) (EuroClone, Pero, Italy) supplemented with 10% of fetal bovine serum (EuroClone, Pero, Italy), 2 mM glutamine (EuroClone, Pero, Italy), 100 U/mL penicillin, and 100 mg/mL streptomycin (EuroClone, Pero, Italy).

### 4.2. ACE2 Expression in HMEC-1 and in VERO E6 Cells 

Cell protein extraction was performed from 1 × 106 HMEC-1 and VERO E6, using TNTG buffer (50 mM Tris-HCl pH 7.5, 150 mM NaCl, 100 mM NaF, 10 mM sodium pyrophosphate, 10% (*v*/*v*) glycerol, 1% Triton X–100), supplemented with complete Mini Protease Inhibitor Cocktail (Merck, Darmstadt, Germany) and 2 mM activated orthovanadate (Merck, Germany). Insoluble material was removed via 10 min of centrifugation at 16,200× *g*, at 4 °C. Protein concentration was determined by Coomassie Plus (Bradford, UK) Assay Reagent (ThermoFisher Scientific, Waltham, MA, USA) using Ultrospec 2100 pro spectrophotometer (GE Healthcare, Chicago, IL, USA). A 30 µg amount of total protein was loaded and electrophoretically separated on NuPAGE™ 4 to 12%, Bis-Tris Mini Protein Gel (ThermoFisher Scientific, Waltham, MA, USA). Separated proteins were electrophoretically transferred onto Immobilon-P Transfer membrane (Merck, Darmstadt, Germany) for 2 h at constant 250 mA at 4 °C. Subsequently, the membrane was stained with Ponceau S solution (Merck, Darmstadt, Germany) to check protein loading, washed extensively with TBS (50 mM Tris, 150 mM NaCl, pH 7.4) + 0.1% Tween-20 (Merck, Darmstadt, Germany), and saturated for 1 h in blocking solution (5% low-fat milk (Genespin, Milan, Italy) in TBS + 0.1% Tween-20) at room temperature. At the end of the saturation, the membrane was incubated with ACE2 Recombinant Rabbit Monoclonal Antibody (dilution 1:1000) (ThermoFisher Scientific, Waltham, MA, USA) in 5% low-fat milk in TBS + 0.1% Tween-20 overnight at 4 °C, with gentle shaking. The membrane was then washed with TBS + 0.1% Tween-20 and further incubated with horseradish peroxidase (HRP)-conjugated anti-rabbit secondary antibody (dilution 1:10,000) (Amersham GE Healthcare, Amersham, UK) for 1 h at room temperature. β-actin served as loading control. To detect β-actin, the membrane was incubated with monoclonal Anti-β-Actin peroxidase-linked antibody (dilution 1:30,000) (Merck, Darmstadt, Germany) in 5% low-fat milk in TBS + 0.1% Tween-20 for 1 h at 4 °C, with gentle shaking. Signals were detected using the enhanced chemiluminescence method (ECL, Amersham GE Healthcare, Amersham, UK), performed according to the manufacturer’s instructions.

### 4.3. SARS-CoV-2 Infection

A total of 1.5 × 10^5^ HMEC-1 or VERO E6 cells/well were seeded in a 24-well culture multi-well plate and left to adhere overnight in complete medium at 37 °C, in a humidified atmosphere containing 5% CO_2_, until 80% confluence was reached. Cell infection was obtained by viral adsorption in complete medium using a Multiplicity of infection (MOI) of 0.1 (8.78 × 10^8^ copies/mL) of SARS-CoV-2 viral strains B.1 (SARS-CoV-2/human/ITA/Milan-UNIMI-1/2020, Gen Bank accession ID: MT748758.1), B.1.617.2 (GISAID EPI_ISL_6602889), and B.1.1.529, BA.1 (EPI_ISL_10898045), previously isolated in our laboratory [41], for 2 h at 37 °C, in a humidified atmosphere containing 5% CO_2_. After 72 h p.i., the presence and localization of SARS-CoV-2 virions was evaluated by Transmission Electron Microscopy (TEM); furthermore, SARS-CoV-2 specific one-step Quantitative Reverse Transcriptase Real Time Polymerase Chain Reaction (qRT-PCR) on cell supernatant and cellular RNA of infected cell after 4, 6, 24, 48, 72 h and 7 days p.i. was performed.

All studies with SARS-CoV-2 were performed in a Biosafety Level 3 laboratory. 

### 4.4. Transmission Electron Microscopy (TEM)

Analysis of SARS-CoV-2 presence and localization in infected HMEC-1 cells was performed using TEM by Unitech NOLIMITS, Università degli Studi di Milano. Cells were infected at MOI of 0.5 and cultured for 7 days. After washing once with PBS Dulbecco’s Phosphate Buffered Saline (DPBS), cell monolayers were fixed with glutaraldehyde 2.5% and incubated at room temperature for 30 min. Cells were then scraped off. The fixation buffer containing cell suspension was transferred into a tube and subjected to centrifugation for 10 min at 12,000× *g*. The tube was stored at 4 °C for at least 24 h before preparation for TEM analysis.

### 4.5. RNA Extraction

At 4, 6, 24, 48, 72 h and 7 days p.i., supernatants and cells were harvested (non-infected -mock- and infected HMEC-1, and VERO E6 cells) and RNA was extracted using RNA Blood mini kit (Qiagen, Hilden, Germany) and Nucleospin RNA virus Kit (Macherey Nagel, Düren, Germany), respectively, following the manufacturer’s protocols.

### 4.6. Quantitative-Real Time PCR (qRT-PCR) for SARS-CoV-2 

The presence of SARS-CoV-2 N1 gene in both supernatants and cells was evaluated by means of AgPath-ID One-Step RT-PCR assay (ThermoFisher, Waltham, MA, USA), using the 7500 Real Time PCR system (ThermoFisher Scientific, Waltham, MA, USA). The primer and probe sequences, targeting N1 gene, have been published by CDC and WHO [42,43]. The reaction mix was conducted in a final volume of 25 μL, containing 1× RT-PCR buffer (2×), 0.4 μM of each primer, 0.1 μM of probe, 1× RT-PCR Enzyme mix (25×), and 5 μL of heat-inactivated cell medium. The standard curve was constructed using a serially diluted plasmid pEX-A128-nCoV_all (Eurofins, Luxemburg), containing part of the SARS-CoV-2 genome (3 × 10^7^–3 × 10^1^ copies/μL). Samples were analyzed in duplicate, and a negative control was added. The limit of detection was three copies per reaction. 

To confirm SARS-CoV-2 active replication, SARS-CoV-2 subgenomic RNA (sgRNA) was amplified by means of AgPath-ID One-Step RT-PCR assay, using sgLeadSARSCoV2-F primer, instead of the previous N1-F. The sequence of this primer was the following: sgLeadSARSCoV2-F 5′-CGATCTCTTGTAGATCTGTTCTC-3′ [44]. Calculation of ΔCt between genomic RNA (gRNA) and sgRNA was performed at each time point in infected cells as follows: ΔCt = Ct (sgRNA) − Ct (gRNA).

### 4.7. Cytokine and Human Tissue Factor (hTF) Transcription

A total of 250 ng of isolated cellular RNA was reverse transcribed into cDNA with QuantiTect Reverse Transcription Kit (Qiagen, Hilden, Germany), after DNA removal. IL-6, IL-8, TNF-α, IFN-α, and hTF expression levels were evaluated in SARS-CoV-2 infected and non-infected HMEC-1 by Real Time PCR. Complementary DNA (cDNA) was amplified using QuantiTect SYBR Green PCR Kit (Qiagen, Hilden, Germany) on Applied Biosystem 7500 Real Time PCR system (Applied Biosystem, Waltham, MA, USA). The expression of the cytokines and hTF was normalized to the Glyceraldehyde-3-Phosphate Dehydrogenase (GAPDH) gene expression. The reaction mix was conducted in a final volume of 25 µL, containing 1× QuantiTect SYBR Green (2×), 0.3 µM of each primer, and 2 µL of cDNA. Primer sequences are reported in Table 1. 

The thermal profile consisted of 2 min at 50 °C, 15 min at 95 °C, and 45 cycles of three steps (15 s at 94 °C; 30 s at 60 °C for IL-6, IL-8, TNF- α, hTF, and GAPDH or 30 s at 54 °C for IFN-α; and 30 s at 72 °C). All Samples were analyzed in duplicate. Quantification of gene expression was obtained using Fold Difference.

ΔCt cytokines = Ct cytokines − Ct GAPDH
ΔCt mean cytokines = ∑Ct cytokines number of samples
ΔΔCt = ΔCt mean cytokines in infected cells − ΔCt mean cytokines in non-infected cells
Fold Difference = 2^−ΔΔCt^

### 4.8. IL-6, IL-8, TNF-α, and IFN-α Titres

Interleukin and cytokine production was determined in infected and non-infected cell supernatants, using the following ELISA commercial kits, in accordance with the manufacturer’s instructions: Human IL-6 DuoSet ELISA (R&D Systems, Minneapolis, MN, USA) for IL-6, Human IL-8/CXCL8 DuoSet ELISA (R&D Systems, Minneapolis, MN, USA) for IL-8, Human IFN-alpha all Subtype Quantikine ELISA Kit (R&D Systems, Minneapolis, MN, USA) for IFN-α. Concentrations are expressed as pg/mL of cell culture medium. 

### 4.9. Statistical Analysis

ACE2 expression in VERO E6 and HMEC-1 cells was evaluated using ImageJ, GraphPad Prism, and paired Student’s *t*-tests. SARS-CoV-2 qRT-PCR results were analyzed by the absolute quantification method and are expressed as viral copies/mL of cell supernatants, and viral copies/µg for cellular RNA. Cytokine concentrations in cell mediums were evaluated by the ELISA method and are expressed as pg/mL. For statistical comparison, significance was evaluated by means of Multiple *t*-test using the two-stage linear step-up procedure of Benjamini, Krieger, and Yekutieli, with Q = 1% for interleukin and cytokine production and with two-way ANOVA test with multiple comparisons for cytokine and hTF transcription levels. Values of *p* < 0.001 (***) and *p* < 0.05 (*) were considered statistically significant.

## Figures and Tables

**Table 1:** SYBR Green Real Time PCR primer sequences.

Target Gene	Forward Primer (5′-3′)	Reverse Primer (5′-3′)
*IL-6* [45]	GTAGCCGCCCCACACAGACAGCC	GCCATCTTTGGAAGGTTC
*IL-8* [46]	CCACCGGAAGGAACCATCTC	GGGGTGGAAAGGTTTGGAGT
*TNF-α* [47]	CTCCAGGCGGTGCCTATGT	GAAGAGCGTGGTGGCCC
*IFN-α* [46]	AGAATCACTCTCTATCTGAAAGAGAAG	TCATGATTTCTGCTCTGACAACCT
*hTF* [48]	TCCCCAGAGTTCACACCTTACC	TGACCACAAATACCACAGCTCC
*GAPDH* [49]	GCCCAGGATGCCCTTGA	GTGTCCCCACTGCCAAC

## Data Availability

All datasets generated for this study are included in the article. Genome sequence with detailed information of the strains is available in the GISAID database, with the accession numbers reported in the text.

## Supplementary Materials

The following supporting information can be downloaded at https://www.mdpi.com/article/10.3390/ijms23074063/s1.

## References

[1] Hikmet F., Méar L., Edvinsson Å., Micke P., Uhlén M., Lindskog C. (2020). The protein expression profile of ACE2 in human tissues. Mol. Syst. Biol..

[2] Ortiz-Prado E., Simbaña-Rivera K., Gómez-Barreno L., Rubio-Neira M., Guaman L.P., Kyriakidis N.C., Muslin C., Jaramillo A.M.G., Barba-Ostria C., Cevallos-Robalino D. (2020). Clinical, molecular, and epidemiological characterization of the SARS-CoV-2 virus and the Coronavirus Disease 2019 (COVID-19), a comprehensive literature review. Diagn. Microbiol. Infect. Dis..

[3] Zhou P., Yang X.L., Wang X.G., Hu B., Zhang L., Zhang W., Si H.R., Zhu Y., Li B., Huang C.L. (2020). A pneumonia outbreak associated with a new coronavirus of probable bat origin. Nature.

[4] Cooke J. (2000). The endothelium: A new target for therapy. Vasc. Med..

[5] Varga Z., Flammer A., Steiger P., Haberecker M., Andermatt R., Zinkernagel A., Mehra M., Schuepbach R., Ruschitzka F., Moch H. (2020). Endothelial cell infection and endotheliitis in COVID-19. Lancet.

[6] Schaefer I.M., Padera R.F., Solomon I.H., Kanjilal S., Hammer M.M., Hornick J.L., Sholl L.M. (2020). In situ detection of SARS-CoV-2 in lungs and airways of patients with COVID-19. Mod. Pathol..

[7] Sardu C., Gambardella J., Morelli M.B., Wang X., Marfella R., Santulli G. (2020). Hypertension, thrombosis, kidney failure, and diabetes: Is covid-19 an endothelial disease? a comprehensive evaluation of clinical and basic evidence. J. Clin. Med..

[8] Hojyo S., Uchida M., Tanaka K., Hasebe R., Tanaka Y., Murakami M., Hirano T. (2020). How COVID-19 induces cytokine storm with high mortality. Inflamm. Regen..

[9] Jin Y., Ji W., Yang H., Chen S., Zhang W., Duan G. (2020). Endothelial activation and dysfunction in COVID-19: From basic mechanisms to potential therapeutic approaches. Signal Transduct. Target. Ther..

[10] Tang Y., Liu J., Zhang D., Xu Z., Ji J., Wen C. (2020). Cytokine Storm in COVID-19: The Current Evidence and Treatment Strategies. Front. Immunol..

[11] Colmenero I., Santonja C., Alonso-Riaño M., Noguera-Morel L., Hernández-Martín A., Andina D., Wiesner T., Rodríguez-Peralto J.L., Requena L., Torrelo A. (2020). SARS-CoV-2 endothelial infection causes COVID-19 chilblains: Histopathological, immunohistochemical and ultrastructural study of seven paediatric cases. Br. J. Dermatol..

[12] Caccuri F., Bugatti A., Zani A., De Palma A., Di Silvestre D., Manocha E., Filippini F., Messali S., Chiodelli P., Campisi G. (2021). Sars-cov-2 infection remodels the phenotype and promotes angiogenesis of primary human lung endothelial cells. Microorganisms.

[13] Monteil V., Kwon H., Prado P., Hagelkrüys A., Wimmer R.A., Stahl M., Leopoldi A., Garreta E., Hurtado del Pozo C., Prosper F. (2020). Inhibition of SARS-CoV-2 Infections in Engineered Human Tissues Using Clinical-Grade Soluble Human ACE2. Cell.

[14] Schimmel L., Chew K.Y., Stocks C.J., Yordanov T.E., Essebier P., Kulasinghe A., Monkman J., Santos Miggiolaro A.F.R., Cooper C., Noronha L. (2021). Endothelial cells are not productively infected by SARS-CoV-2. Clin. Transl. Immunol..

[15] Wurtz N., Penant G., Jardot P., Duclos N., La Scola B. (2021). Culture of SARS-CoV-2 in a panel of laboratory cell lines, permissivity, and differences in growth profile. Eur. J. Clin. Microbiol. Infect. Dis..

[16] Ferrario C.M., Jessup J., Chappell M.C., Averill D.B., Brosnihan K.B., Tallant E.A., Diz D.I., Gallagher P.E. (2005). Effect of angiotensin-converting enzyme inhibition and angiotensin II receptor blockers on cardiac angiotensin-converting enzyme 2. Circulation.

[17] Ashraf U.M., Abokor A.A., Edwards J.M., Waigi E.W., Royfman R.S., Hasan S.A.M., Smedlund K.B., Hardy A.M.G., Chakravarti R., Koch L.G. (2021). Sars-cov-2, ace2 expression, and systemic organ invasion. Physiol. Genom..

[18] Cantuti-Castelvetri L., Ojha R., Pedro L.D., Djannatian M., Franz J., Kuivanen S., van der Meer F., Kallio K., Kaya T., Anastasina M. (2020). Neuropilin-1 facilitates SARS-CoV-2 cell entry and infectivity. Science.

[19] Daly J.L., Simonetti B., Klein K., Chen K.E., Williamson M.K., Antón-Plágaro C., Shoemark D.K., Simón-Gracia L., Bauer M., Hollandi R. (2020). Neuropilin-1 is a host factor for SARS-CoV-2 infection. Science.

[20] Raimondi C., Brash J.T., Fantin A., Ruhrberg C. (2016). NRP1 function and targeting in neurovascular development and eye disease. Prog. Retin. Eye Res..

[21] Soker S., Takashima S., Miao H.Q., Neufeld G., Klagsbrun M. (1998). Neuropilin-1 is expressed by endothelial and tumor cells as an isoform- specific receptor for vascular endothelial growth factor. Cell.

[22] Matheson N.J., Lehner P.J. (2020). How does SARS-CoV-2 cause COVID-19?. Science.

[23] Hoffmann M., Kleine-Weber H., Schroeder S., Krüger N., Herrler T., Erichsen S., Schiergens T.S., Herrler G., Wu N.H., Nitsche A. (2020). SARS-CoV-2 Cell Entry Depends on ACE2 and TMPRSS2 and Is Blocked by a Clinically Proven Protease Inhibitor. Cell.

[24] Shang J., Wan Y., Luo C., Ye G., Geng Q., Auerbach A., Li F. (2020). Cell entry mechanisms of SARS-CoV-2. Proc. Natl. Acad. Sci. USA.

[25] Aimes R.T., Zijlstra A., Hooper J.D., Ogbourne S.M., Sit M.L., Fuchs S., Gotley D.C., Quigley J.P., Antalis T.M. (2003). Endothelial cell serine proteases expressed during vascular morphogenesis and angiogenesis. Thromb Haemost..

[26] Pantazi I., Al-Qahtani A.A., Alhamlan F.S., Alothaid H., Matou-Nasri S., Sourvinos G., Vergadi E., Tsatsanis C. (2021). SARS-CoV-2/ACE2 Interaction Suppresses IRAK-M Expression and Promotes Pro-Inflammatory Cytokine Production in Macrophages. Front. Immunol..

[27] Patra T., Meyer K., Geerling L., Isbell T.S., Hoft D.F., Brien J., Pinto A.K., Ray R.B., Ray R. (2020). SARS-CoV-2 spike protein promotes IL-6 transsignaling by activation of angiotensin II receptor signaling in epithelial cells. PLoS Pathog..

[28] Liu Y., Zhang C., Huang F., Yang Y., Wang F., Yuan J., Zhang Z., Qin Y., Li X., Zhao D. (2020). Elevated plasma levels of selective cytokines in COVID-19 patients reflect viral load and lung injury. Natl. Sci. Rev..

[29] Pedersen S.F., Ho Y.C. (2020). SARS-CoV-2: A storm is raging. J. Clin. Investig..

[30] McElvaney O.J., McEvoy N.L., McElvaney O.F., Carroll T.P., Murphy M.P., Dunlea D.M., Choileáin O.N., Clarke J., O’Connor E., Hogan G. (2020). Characterization of the inflammatory response to severe COVID-19 Illness. Am. J. Respir. Crit. Care Med..

[31] Karwaciak I., Sałkowska A., Karaś K., Dastych J., Ratajewski M. (2021). Nucleocapsid and spike proteins of the coronavirus SARS-CoV-2 induce il6 in monocytes and macrophages—Potential implications for cytokine storm syndrome. Vaccines.

[32] Zhang X., Wu K., Wang D., Yue X., Song D., Zhu Y., Wu J. (2007). Nucleocapsid protein of SARS-CoV activates interleukin-6 expression through cellular transcription factor NF-κB. Virology.

[33] Kang S., Kishimoto T. (2021). Interplay between interleukin-6 signaling and the vascular endothelium in cytokine storms. Exp. Mol. Med..

[34] Imaizumi T., Itaya H., Fujita K., Kudoh D., Kudoh S., Mori K., Fujimoto K., Matsumiya T., Yoshida H., Satoh K. (2000). Expression of tumor necrosis factor-alpha in cultured human endothelial cells stimulated with lipopolysaccharide or interleukin-1alpha. Arter. Thromb Vasc. Biol..

[35] Ranta V., Orpana A., Carpén O., Turpeinen U., Ylikorkala O., Viinikka L. (1999). Human vascular endothelial cells produce tumor necrosis factor-alpha in response to proinflammatory cytokine stimulation. Crit. Care Med..

[36] Kraus A.A., Raftery M.J., Giese T., Ulrich R., Zawatzky R., Hippenstiel S., Suttorp N., Krüger D.H., Schönrich G. (2004). Differential Antiviral Response of Endothelial Cells after Infection with Pathogenic and Nonpathogenic Hantaviruses. J. Virol..

[37] Flammer A.J., Anderson T., Celermajer D.S., Creager M.A., Deanfield J., Ganz P., Hamburg N.M., Lüscher T.F., Shechter M., Taddei S. (2012). The assessment of endothelial function: From research into clinical practice. Circulation.

[38] Subramaniam S., Scharrer I. (2018). Procoagulant activity during viral infections. Front. Biosci.

[39] Bautista-Vargas M., Bonilla-Abadía F., Cañas C.A. (2020). Potential role for tissue factor in the pathogenesis of hypercoagulability associated with in COVID-19. J. Thromb. Thrombolysis.

[40] Ades E., Candal F., Swerlick R., George V., Summers S., Bosse D., Lawley T. (1992). HMEC-1: Establishment of an immortalized human microvascular endothelial cell line. J. Investig. Dermatol..

[41] Delbue S., D’Alessandro S., Signorini L., Dolci M., Pariani E., Bianchi M., Fattori S., Modenese A., Galli C., Eberini I. (2021). Isolation of SARS-CoV-2 strains carrying a nucleotide mutation, leading to a stop codon in the ORF 6 protein. Emerg. Microbes Infect..

[42] Centers for Disease Control and Prevention (CDC). https://www.cdc.gov/.

[43] World Health Organization (WHO). https://www.who.int/.

[44] Moreira L., de Souza Luna L., Barbosa G., Perosa A., Chaves A., Conte D., Carvalho J., Bellei N. (2021). Test on stool samples improves the diagnosis of hospitalized patients: Detection of SARS-CoV-2 genomic and subgenomic RNA. J. Infect..

[45] Strong A.L., Gimble J.M., Bunnell B.A. (2015). Analysis of the Pro- and Anti-Inflammatory Cytokines Secreted by Adult Stem Cells during Differentiation. Stem Cells Int..

[46] Shuai H., Chu H., Hou Y., Yang D., Wang Y., Hu B. (2020). Differential immune activation profile of SARS-CoV-2 and SARS-CoV infection in human lung and intestinal cells: Implications for treatment with IFN- β and IFN inducer. Br. J. Dermatol..

[47] Wilsmann-Theis D., Koch S., Mindnich C., Bonness S., Schnautz S., Von Bubnoff D., Bieber T. (2013). Generation and functional analysis of human TNF-α/iNOS-producing dendritic cells (Tip-DC). Allergy Eur. J. Allergy Clin. Immunol..

[48] Cañas C., Cañas F., Bautista-Vargas M., Bonilla-Abadía F. (2021). Role of Tissue Factor in the Pathogenesis of COVID-19 and the Possible Ways to Inhibit It. Clin. Appl. Thromb..

[49] Rhyu D.-W., Kang Y.-J., Ock M.-S., Eo J.-W., Choi Y.-H., Kim W.-J., Leem S.-H., Yi J.-M., Kim H.-S., Cha H.-J. (2014). Expression of Human Endogenous Retrovirus env Genes in the Blood of Breast Cancer Patients. Int. J. Mol. Sci..

