# Supplementary material for: In Vitro SARS-CoV-2 Infection of Microvascular Endothelial Cells: Effect on Pro-Inflammatory Cytokine and Chemokine Release
Source: Int J Mol Sci. 2022 Apr 6;23(7):4063. doi: 10.3390/ijms23074063 (PMC8999888; doi:10.3390/ijms23074063)
Supplement: Supplementary file 1 [file ijms-23-04063-s001.zip › ijms-1579107-supplementary.pdf]

International Journal of *Molecular Sciences*

MDPI

Table S1. SARS-CoV-2 N1 gene expression in HMEC-1 and VERO E6 supernatant and cellular RNA for each viral strain

		Supernatant (copies/mL)						Cellular RNA (copies/µg)					
		**4h**	**6h**	**24h**	**48h**	**72h**	**7 days**	**4h**	**6h**	**24h**	**48h**	**72h**	**7 days**
HMEC-1	B.1	8.11×10^{6}	1.09×10^{7}	7.44×10^{6}	6.23×10^{6}	7.70×10^{6}	7.45×10^{6}	9.12×10^{5}	9.37×10^{5}	6.66×10^{5}	2.92×10^{5}	2.55×10^{5}	1.66×10^{5}
	B.1.617.2	3.03×10^{7}	3.34×10^{7}	4.51×10^{7}	-	-	-	9.30×10^{7}	1.31×10^{8}	6.66×10^{7}	-	-	-
	BA.1-like	1.39×10^{7}	2.26×10^{7}	1.49×10^{7}	-	-	-	7.05×10^{5}	5.73×10^{5}	3.08×10^{5}	-	-	-
VERO E6	B.1	1.33×10^{5}	1.16×10^{5}	1.15×10^{9}	6.03×10^{9}	9.80×10^{9}	2.91×10^{9}	3.52×10^{5}	5.01×10^{6}	1.09×10^{11}	1.85×10^{10}	4.74×10^{9}	2.54×10^{9}
	B.1.617.2	2.21×10^{6}	1.45×10^{6}	3.08×10^{8}	-	-	-	5.44×10^{7}	7.62×10^{7}	3.97×10^{10}	-	-	-
	BA.1-like	4.67×10^{5}	7.04×10^{5}	1.34×10^{7}	-	-	-	5.64×10^{5}	1.51×10^{7}	1.22×10^{9}	-	-	-

Table S2. SARS-CoV-2 N1 gene gRNA and sgRNA Ct values and relative ΔCt values for each viral strain

		gRNA						sgRNA						ΔCt (sgRNA-gRNA)					
		**4h**	**6h**	**24h**	**48h**	**72h**	**7 days**	**4h**	**6h**	**24h**	**48h**	**72h**	**7 days**	**4h**	**6h**	**24h**	**48h**	**72h**	**7 days**
HMEC-1	B.1	23.68	23.94	23.86	24.71	24.92	25.51	27.40	28.10	28.26	29.60	29.00	30.62	3.72	4.17	4.40	4.88	4.09	5.11
	B.1.617.2	18.50	16.96	17.96	-	-	-	20.54	19.35	20.06	-	-	-	2.49	2.39	2.10	-	-	-
	BA.1-like	24.21	24.70	24.41	-	-	-	27.55	27.88	25.62	-	-	-	3.34	3.18	1.20	-	-	-
VERO E6	B.1	-	-	6.78	9.42	10.63	10.73	-	-	9.11	12.66	13.08	12.94	-	-	2.34	3.24	2.45	2.21
